# PAX5 fusion genes in acute lymphoblastic leukemia: A literature review

**DOI:** 10.1097/MD.0000000000033836

**Published:** 2023-05-17

**Authors:** Fatma Mohamed Fouad, Jehane I. Eid

**Affiliations:** a Biology Department, College of Science, Sultan Qaboos University, Muscat, Oman; b Chemistry Department, Biotechnology/Bimolecular Chemistry program, Faculty of Science, Cairo University, Giza, Egypt; c Zoology Department, Faculty of Science, Cairo University, Giza, Egypt.

**Keywords:** ALL, B-ALL, fusion, PAX5

## Abstract

Acute lymphoblastic leukemia (ALL) is a common cancer affecting children worldwide. The development of ALL is driven by several genes, some of which can be targeted for treatment by inhibiting gene fusions. PAX5 is frequently mutated in ALL and is involved in chromosomal rearrangements and translocations. Mutations in PAX5 interact with other genes, such as ETV6 and FOXP1, which influence B-cell development. PAX5/ETV6 has been observed in both B-ALL patients and a mouse model. The interaction between PAX5 and FOXP1 negatively suppresses the Pax5 gene in B-ALL patients.

Additionally, ELN and PML genes have been found to fuse with PAX5, leading to adverse effects on B-cell differentiation. ELN-PAX5 interaction results in the decreased expression of LEF1, MB1, and BLNK, while PML-PAX5 is critical in the early stages of leukemia. PAX5 fusion genes prevent the transcription of the PAX5 gene, making it an essential target gene for the study of leukemia progression and the diagnosis of B-ALL.

## 1. Introduction

Acute lymphoblastic leukemia (ALL) is a malignant disease that develops from the lymphoid precursor cells in the bone marrow. Over a third (34.1%) of all childhood cancer disability-adjusted life years are attributed to ALL, making it the most common childhood cancer worldwide.^[1]^ Research suggests that the highest likelihood of ALL and its pre-B subtype is in children under 5 years old, with the risk declining until the mid-twenties, which starts to increase again.^[2]^ At the mechanistic level, mutations that arise during the differentiation of blood cells are responsible for the onset of ALL.^[3]^ Consequently, these DNA mutations in white blood cells lead to their uncontrollable growth and proliferation. This change can be passed on genetically from parent to child,^[4]^ or it can arise accidentally due to exposure to hazardous substances.^[5,6]^

Moreover, several inherited disorders have been linked to ALL, including Down syndrome,^[7]^ Swyer syndrome,^[8]^ and Bainbridge-Ropers syndrome.^[9]^ It has been indicated earlier that there is a correlation between numerous germline alleles, variants passed down from parent to child, and somatic mutations in children with ALL.^[10,11]^ Several alleles have been linked to ALL, including IKZF1,^[12]^ PAX5,^[13]^ and ETV6^[14]^ in the germline, and KRAS, NRAS, PTPN11, JAK2, and FLT3^[15,16]^ in the somatic mutations.

Despite remarkable progress in treating ALL over the past decades, the disease remains a significant challenge in oncology. This challenge is due to its heterogeneity, with diverse clinical and biological characteristics among ALL patients.^[17]^ ALL have 2 primary subtypes, B- and T-ALL, which have recently been further classified based on whole transcriptome analysis and gene expression grouping using contemporary clinical, cytogenetic, and molecular data.^[18]^ Among ALL subtypes, those originating from the B-cell lineage are the most prevalent, accounting for approximately 85% of cases.^[19]^ However, new B-ALL and T-ALL subtypes have recently been identified, some of which are defined by unique gene rearrangements or a single gene mutation.^[20]^ These novel subtypes have demonstrated distinct clinical features and responses to treatment.

The B-ALL subtype, a prevalent hematologic neoplasm characterized by the uncontrolled proliferation of immature B cells, includes the precursor B-ALL subtype, with the pre-B ALL subtype being the most common form of ALL and predominantly affecting children under the age of 5.^[21]^ Although the risk of developing ALL decreases with age in children, it resurfaces in the mid-twenties, with B-ALL being the most common subtype accounting for 80% of acute leukemia cases in children compared to only 20% in adults. Although B-ALL is one of the leading causes of cancer-related death in children, they have a better chance of surviving than adults.^[22]^ The response to treatment is influenced by the type of genomic alterations, indicating that ALL may have different mechanisms in childhood and adulthood.

Despite the high incidence of ALL in children, adult ALL patients have a poorer prognosis, possibly due to the differences in genetic alterations between the 2 age groups.^[23]^ Identifying new ALL subtypes with unique gene mutations and rearrangements have improved our understanding of the underlying biology of the disease and has led to the development of targeted therapies that have shown promising results in clinical trials.^[24,25]^ However, further research is still needed to fully comprehend the genetic and molecular mechanisms underlying ALL and develop more effective therapies for patients.

The extent to which a patient responds to treatment for leukemic malignancies is influenced by the type of genomic alterations screened.^[26]^ In many childhoods, ALL cases do not have a recurrent chromosomal abnormality, despite the possibility of recurrent genetic abnormalities. The genetic propensity of these cases has yet to be fully understood at this time.^[27,28]^

Precursor B-cell acute lymphoblastic leukemia (B-ALL) has been the focus of intense research in recent years due to the discovery of genetic subtypes with distinct biomarker ramifications. ALL is a complex illness with various risk factors, including genetic predisposition. Distinct recurrent genetic abnormalities can be used to identify ALL phenotypic subgroups; they are crucial for clinical specification and, in some cases, risk grading and precision medicine. It has been discovered that there are over 20 different subtypes of B-ALL, each with its own distinctive risk of relapse.^[29]^ A study uncovered adult B-ALL’s genetic and molecular architecture, revealing 2 distinct variants that aid in diagnosing patients with Swyer syndrome^[8]^ and Bainbridge.^[9]^ For this review, we will focus specifically on the role of PAX5 mutations in the development of ALL.

The PAX5 gene, located at 9p13, is a vital transcription factor protein that belongs to the paired box (Pax) family. This family comprises 9 DNA-binding domains crucial in organogenesis and brain development. PAX5 is implicated in the pathogenesis of many cases of ALL,^[30]^ including somatic and germline mutations. It is imperative in the early stages of B cell lineage development, where it plays a role in the commitment and maintenance of B-cells in childhood B-ALL.^[31]^ However, PAX5 is frequently affected by various mutations, including deletions, amplifications, rearrangements, and point mutations.^[32–36]^ The expression of PAX5 is mainly associated with B lymphocyte maturation, cell survival, motility, and tumor progression, and it varies as cancer progresses. This review will primarily focus on PAX5 mutations and their implications in ALL development.

The PAX5 gene is a paired box (Pax) family member, a domain of 9 highly conserved DNA-binding domains involved in organogenesis and brain development.^[37–40]^ PAX5 plays a crucial role in embryonic development and cell differentiation.^[41]^ It is expressed from the pro-B to the mature B-cell stage in the bone marrow and is downregulated in the final stage of differentiation into plasma cells.^[42]^ PAX5 is the primary target of genetic alterations in B-ALL, with more than a third of patients having mutations that result in downregulation and a defect in DNA-binding activity and expression. Additionally, approximately 2.6% of pediatric B-ALL patients have a rearrangement in PAX5 that fuses it to other genes,^[31,32,35,43]^ causing B-cell development to be blocked, as seen in PAX5-ETV6 and PAX5-FOXP1 fusions. All fusion genes with PAX are conserved in the paired box, such as PAX5-ETV6, PAX3-FKHR, and PAX8-PPARG. Although almost 80% of childhood cancer cases are curable, many pediatric patients resist therapy, leading to poor outcomes.^[44]^

Furthermore, adult ALL cases have a poor prognosis,^[45]^ with a relapse rate of 40.0% compared to 9.6% in pediatrics.^[46]^ Treating ALL remains challenging due to the high risk of relapse shortly after complete remission,^[46]^ which often results in refractory leukemia that is difficult to cure, with a 5-year overall survival rate of approximately 50%.^[45]^ This review will focus on the implications of PAX5 mutations in ALL development, particularly B-ALL. Table 1 briefly overviews the review’s focus on the most common fusion occurring with PAX5 in ALL.

**Table 1:** Fusion between PAX5 and other genes in acute lymphoblastic leukemia.

Fusion genes	Position	Number of cases	Genes affected	Disease	References
PAX5/ETV6	dic (9;12) (p13;p3)	3	BLNK suppressed	B-ALL	Bousquet M. 2007, Chen Y. 2022, Ha J. 2018^[38,44,47]^
PAX5-ELN	t (7;9) (q11;p13)	2	*LEF1, BLNK*, and *MB1down regulated*	B-ALL	Nutt SL. 1998^[37]^
PAX5-FOXP1	t (3;9) (p13;p13)	1	PAX5 is suppressed	B-ALL	Familiades J. 2009, Strehl S. 2003^[28,48]^
	t (3;9) (p14;p13)	1			
PAX5-PML	t (9;15) (p21;q25)	1	PAX5 and PML inhibited	ALL	Put N. 2011, Nebral K. 2007^[49,50]^
	t (p13;q24) (9;15)	1			

ALL = acute lymphoblastic leukemia, B-ALL = B-cell acute lymphocytic leukemia, PAX5 = paired box 5.

## 2. Mutations in PAX5

PAX5 is a critical transcription factor that plays an essential role in B cell lineage development and is also linked to the development of ALL.^[51]^ The mutations most commonly associated with PAX5 in ALL, particularly B-ALL, result in downregulation and impair DNA-binding activity and expression, contributing to leukemia.^[48,52]^ The article underscores how PAX5 is often subjected to deletions, amplifications, rearrangements, and point mutations. The review summarizes the different mutations related to PAX5 and their implications in ALL development. It emphasizes how they lead to the emergence of refractory leukemia that is challenging to cure, with a 5-year overall survival rate of approximately 50%.

### 2.1. Rearrangement

Rearrangements in the PAX5 gene are a common mutation in acute lymphoblastic leukemia (ALL), leading to the fusion of PAX5 with other genes.^[47]^ These rearrangements affect the function of PAX5, resulting in downregulation or a defect in DNA-binding activity and expression, contributing to the development of leukemia.^[52]^ The most frequent rearrangements are with the ETV6 and ZNF521 genes, which lead to ETV6-PAX5 and ZNF521-PAX5 fusion proteins, respectively.^[53,54]^ These fusion proteins interfere with normal B cell differentiation and maturation, accumulating leukemic cells, and have altered transcriptional activity, leading to the deregulation of target genes involved in B cell development and differentiation. Other rearrangements in PAX5 have also been observed with genes such as BCR,^[55]^ MLL,^[56]^ and FOXP1,^[57]^ which lead to the formation of fusion proteins that affect the function of PAX5 and contribute to the development of leukemia.^[58]^ Detecting rearrangements in PAX5 is crucial for diagnosing and classifying ALL, risk stratification, and treatment selection. The detection of the ETV6-PAX5 fusion protein, for example, is associated with a poor prognosis and a higher risk of relapse, which may require more intensive therapy.^[59]^ Furthermore, the detection of rearrangements in PAX5 provides a potential therapeutic target for the treatment of ALL. Small molecule inhibitors of the ETV6-PAX5 fusion protein have been developed and have shown promising results in preclinical studies.

### 2.2. Translocation

In ALL, chromosomal translocations involving the PAX5 gene have been identified as significant genetic events.^[52]^ These translocations disrupt the PAX5 gene and lead to the formation of PAX5 fusion genes with partner genes from other chromosomes. These fusion genes encode chimeric proteins, which can impair normal B-cell development and contribute to leukemogenesis.^[60]^ PAX5 translocations have been reported in approximately 2% to 3% of pediatric and adult ALL cases.^[61]^ The prevalence and prognostic impact of PAX5 translocations vary depending on the specific fusion gene and other genetic alterations present.

PAX5 is often involved in translocations along with transcription factors, structural proteins, kinases, and other genes.^[32,34,35]^ A t (9;12) translocation is one of the most common PAX5 translocations and is characterized by the presence of a PAX5/ETV6 fusion gene; almost the entire ETV6 transcription factor is fused to the PAX5 paired domain,^[32–34,53,62,63]^ one of the essential transcription factors that underlie hematopoiesis as well as B cell development.^[64]^ Interestingly, the abnormalities in the PAX5 gene are often related to dicentric chromosomes, particularly dic (9;12), while the exact breakpoints vary widely.^[33,63,65,66]^ Earlier studies found a fusion between PAX5 and ETV6 in 18 of 19 patients (95%) with dic (9;12).^[33]^ The dicentric (9;12) translocation usually exists in B-ALL^[63,65]^ and is closely correlated with PAX5-ETV6 fusion; however, it is also present in other chromosomal alterations, such as t (12;21) that comprise ETV6-RUNX1, demonstrating that these actions are coordinated.^[66,67]^

While PAX5-FOXP1 is found in fewer B-ALL cases, it is still a recurrent translocation that connects almost the entire FOXP1 transcription factor sequence to the N-terminal PAX5 sequence.^[32,34]^ PAX5-ETV6 and PAX5-FOXP1 are examples of chimeric PAX5 fusion proteins, which are multi-domain transcription factors. In addition to their DNA-binding domains (Ets and forkhead domains of ETV6 and FOXP1, respectively), both proteins contain an oligomerization motif (pointed domain for ETV6 or a coiled-coil domain for FOXP1).^[34,63]^ Accordingly, PAX5-ETV6 and other PAX5 fused proteins oligomerize.^[67,68]^ Various PAX5 fusion proteins, including PAX5-ETV6 and PAX5-FOXP1, have been reported to suppress the expression levels of wild-type PAX5 in temporal transfection experiments.^[34,43,67,69]^

## 3. Fusion genes with PAX5

### 3.1. PAX5/ETV6 fusion

ETV6 belongs to the ETS family of transcription factors. Its protein contains a pointed N-terminal domain that interacts with other proteins or with itself and a-terminal DNA-binding domain. This gene is shared in a chromosomal rearrangement that causes leukemia (https://www.ncbi.nlm.nih.gov/gene/2120). In human B-ALLs, PAX5 appears to function as a haploinsufficient tumor suppressor.^[34]^ There needs to be more information about the PAX5 mutations that result from the fusion of PAX5 and other genes.^[32,35]^

**Figure 1.**
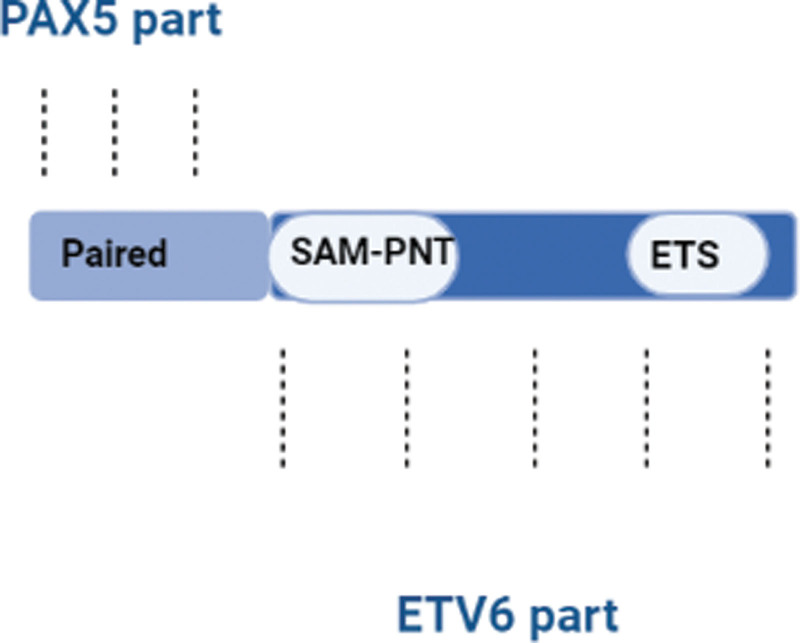
Fusion between PAX5 and ETV6. There is binding between the 3′ end of PAX and 5′ end of ETV6. PAX5 = paired box 5.

PAX5-ETV6 rearrangement was detected in a boy by CMA (chromosomal microarray), and he has pancytopenia, 0.9 × 109/L white blood cells with 30 percent of blasts, he was diagnosed with B-cell ALL (B-ALL). In 8 metaphases, karyotyping reveals 45, XY, 12, and dic (9;12) (p13;p13). The CMA result demonstrated the location and the deletion range, 37 Mbp of deletion on chromosome 9, along with discontinuous 12 and 14 Mbp deletions on chromosome 12. CMA result of fusion detection, a fusion between 3′ of exon 4 of PAX 5 and 5′ of exon 3 of ETV 6 (Fig. 1). This result is also ensured by the RT-PCR and FISH tests.^[53]^

The Pax5-ETV6 complex was studied in mice with B-cell progenitors, but little is known about how these transcription factors regulate gene expression.^[49,50,70,71]^ In addition, analyzing primary human PAX5-ETV6 + B-ALLs can only provide limited insight into the responsibility of PAX5-ETV6 in developing B-ALLs.^[57]^ Therefore, scientists have developed a model for expressing the human protein PAX5-ETV6 in mice. This fusion influences the development of B-cell.^[57]^

The PAX5-ETV6 protein is a dominant negative protein^[34]^; it has a DNA-binding region (Ets [ETV6] domain), a motif (pointed [ETV6], and PAX5 paired domain.^[34,63]^ PAX5-ETV6 in pro-B cells could suppress and activate a few of the activated and repressed PAX5. This fusion also regulates different genes, so the fusion gene with PAX5 controls its job. PAX5-ETV6 and PAX5 interconnect at the same binding site in the genome, so they compete in the same place.^[57]^

The same study analyzed 9 samples of humans with B-ALLs by RNA-seq. The result was that PAX5-ETV6 translocations appeared in these cases. Four of the 9 PAX5-ETV6 + B-ALLs in humans showed repression of the tumor suppressor gene BLNK. However, another function was added to PAX5 as a strong oncoprotein, a portion of PAX5 fusion proteins in B-ALL.^[57]^

In another study, 2 patients were diagnosed with ALL L1; one was treated with the ALL-BFM2000 protocol, and the second with the ALL-BFM86 protocol. The cytogenetic test revealed t (9;12) (p12;p13) in the first patient and dic (9;12) (p11–13;p11–12) in the second. The abnormalities in 12p influence the ETV6 gene, so the FISH test is used to examine ETV6. The 3′–end of the gene persisted on the dic (9; 12) chromosome, but the 5′-end was removed. The breakpoints on the cytogenetic test indicated that ETV6 may have presumably fused with PAX5. PAX5’s 5′ end has been found on the dic (9; 12) chromosome, but the 3′ end has been removed.^[65]^ RT-PCR sequencing revealed that exon 4 of PAX5 fused with exon 3 of ETV6. In both cases, PAX5 is located at 9p13 and ETV6 at 12p13. So, it should be referred to dic (9; 12)-PAX5/ETV6 as dic (9;12) (p13;p13). Therefore, it is clear that dic (9;12), described as a chromosomal mutation primarily found in pre-B hematopoietic malignancies, occurs due to PAX5-ETV6 fusion.^[65]^

### 3.2. PAX5 and ELN:

ELN encodes one of the elastic fiber proteins, which give tissues such as skin, lungs, and blood vessels elasticity. Elastokines are the products of ELN protein degradation. They induce monocyte proliferation and can promote cancer to progress (https://www.ncbi.nlm.nih.gov/gene/2006).^[72]^

To study the role of PAX5-ELN in B-leukemics, they used a KI mouse to mimic PAX5-ELN in humans. PAX5-ELN affects the expression of a few Pax5-regulated genes in vivo, but oncoproteins cause a dominant-negative effect. Although IgH regulatory factors control PAX5-ELN, the fusion level is lower than PAX5. B-ALL in mice is induced by PAX5-ELN, which deregulated preleukemic pro-B cells, and regulated some different molecular programs in them, including 6 activated genes. These mutations appeared on the RAS/MAPK and JAK/STAT pathways via PAX5-ELN. The test on human patients reveals mutations in KRAS, PTPN11, PAX5, NRAS, and JAK3 in divergent types of B-ALL involving PAX5-ELN B-ALL and leukemia due to PAX5-rearrangement.^[73]^

There was a significant increase in the pro-B cell population when PAX5-ELN was administered. This pro-B cell expansion is related to depletion in immature and circulating B cells in the bone marrow, suggesting that PAX5-ELN prevented B-cell differentiation at the preleukemic stage. In peripheral lymphoid organs, PAX5-ELN maintained B-cell loads. PAX5-ELN oncoprotein promotes the expansion of preleukemic pro-B cells by preventing their normal maturation in vivo. In addition, pro-B cells increased abnormally in the preleukemic phases because of the effect of PAX5-ELN, which may induce leukemia.^[73]^

Another study found 2 cases of B-ALL with a translocation of t (7;9) (q11;p13). The FISH test detected the arrangement in PAX5; results reveal a breakpoint between exons 7 and 10. So PAX5 is fused with another gene. Sequencing demonstrated that the partner gene of PAX5 is ELN.^[43]^

PAX5-ELN and PAX5 were both detected in the nucleus by confocal microscopy. The results of CHIP experiments clearly showed that PAX5-ELN could bind BLK, CD19, and wild-type PAX5 sequences, suggesting a dominant-negative activity. HeLa cells co-transfected with a luc-CD19 construct, pcDNA3-PAX5, and pcDNA3-PAX5-ELN showed a decline in PAX5-driven CD19 transcription compared to the amount of pcDNA3-PAX5-ELN transfected. Consequently, PAX5-ELN could block PAX5-dependent transactivation by interacting with PAX5-binding sites.^[43]^ RQ-PCR was done on DG75 cells to measure the effect of PAX5-ELN on other endogenous genes, revealing that PAX5-ELN down-regulated *LEF1, BLNK*, and *MB1* and did not affect the transcription of *BLK* and *CD19*. PAX5-ELN competes with PAX5, so it may interfere with B-cell differentiation, closely linked to PAX5 expression.^[43]^

### 3.3. PAX5-FOXP1

FOXP1 (Forkhead box protein P1) is one of a group of transcription factors called FOX; their DNA-binding domain is named the forkhead domain or the winged-helix.^[74]^ Several studies indicate that FOX proteins are critical for immune control, regulating the survival of lymphocytes to the development of thymocytes.^[75]^ Furthermore, the FOX transcription factors play a role in carcinogenesis by chromosomal translocation, retroviral integration, gene amplification, and transcriptional regulation.^[76]^ FOXP1 is confirmed by gene expression and immunophenotypic studies; it is regulated on normal B cells but overexpressed on diffuse large B-cell lymphoma subgroups.^[77]^ Mutations in FOXP1 cause a disturbance in the development of B cells because it plays an essential role in regulating B lymphopoiesis.^[78]^

A translocation t (3;9) (p13;p13) was found in a man with B-cell acute lymphoblastic leukemia (B-ALL). His blood count was 37.230 × 109/L with leukocytosis and 60.3% blasts, his LDH level was (1710 IU/L), (47,000/mL) for thrombocytopenia, and his Hemoglobin was normal. A sample from his bone marrow revealed TdT, CD10, CD20, and cyCD79a were positive, while cym, CD3, and myeloperoxidase were negative.^[79]^ In 2/6 and 4/4 metaphases, respectively, and in 164/200 and 175/200 interphase nuclei, his FISH result was ish t (3;9) (p13, p13) (3′FOXP1+, 5′FOXP1+; 3′FOXP1+) and ish t (3;9) (p13, p13) (3′PAX5+; 3′PAX5dim, 5′PAX5+). The forkhead box P1(FOXP1) gene was fused with the paired box 5 (PAX5) gene.^[79]^

Another study used a fusion of PAX5 and FOXP1 at exons 6 and 7 to ensure chimeric transcription in 2 patients with t (3;9) (p14;p13), which RCA-RACE and FISH^[67]^ identified.

The 9p and 3′ portions of PAX5 were deleted in 5 patients with B-progenitor cells. Two of them have a fusion of exon 6 and exon 7 of PAX5 and (FOXP1), respectively, and these results were ensured by RT-PCR, FISH, and sequencing analyses. Mutated Pax5 decreased the activation of transcription, and the transaction process to increase PAX5-FOXP1 and a constant amount of PAX5 wild type showed the fusion proteins blocked the transcription process of PAX5.^[34]^

Only 4 cases of PAX5-FOXP1 translocation have been reported so far. Translocation in 2 was examined by a single nucleotide polymorphism array.^[34,67]^ The translocation in the other 2 females was examined using RCA-RACE.^[32]^ The fusion of PAX5 and FOXP1 inhibits PAX5 activity,^[79]^ which may lead to leukemogenesis by preventing hematopoietic cells from converting into mature B cells.^[34,35,67]^

### 3.4. PAX5-PML

This gene encodes a protein that belongs to the tripartite motif family. It is localized in nuclear bodies, serving as a transcription factor and tumor suppressor. In response to oncogenic signals, this gene regulates p53. It is known to be involved in translocations with the retinoic acid receptor alpha gene, which is related to acute promyelocytic leukemia (https://www.ncbi.nlm.nih.gov/gene/5371).

The cytogenetic test of 2 ALL patients revealed a t (9; 15) in the first and a t (9; 15) (p21; q25) in the second. FISH and hybridization ensured that PAX5 and PML were fused. Accordingly, the karyotype was refined to be t (p13;q24) (9;15) in the first patient, and t (9;15) (p21;q25) in the second.^[80]^ Using primers in Exon 5 of PAX5 and Exon 2 of PML, the RT-PCR test revealed both patients’ chimeric transcripts of PAX5 and PML. PAX5-PML fusion consisted of the PAX5 octapeptide and the partial homeodomain, the paired domain, and the PML protein without a 5′ proline-rich region. Different primer combinations failed to amplify the reciprocal PML-PAX5 fusion transcript, indicating that the PAX5-PML fusion manages the initiation of leukemia.^[80]^

**Figure 2.**
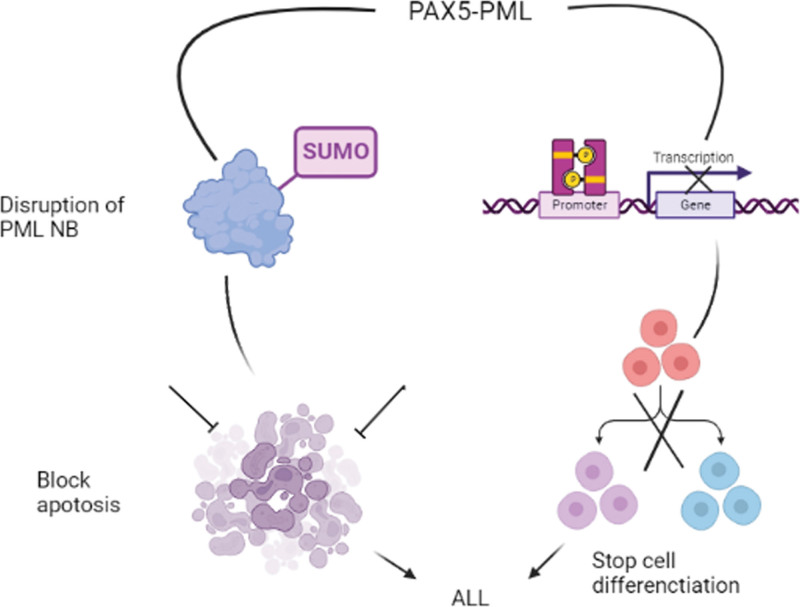
Relationship between ALL (acute lymphoblastic leukemia) and PAX5-PML. Fusion between PAX5 and PML leads to the blocking of transcription and expression of PAX5 and affects the differentiation of B cells. Conversely, PML nuclear bodies are disrupted and inhibit the apoptosis process. PAX5 = paired box 5.

PAX5-PML negatively impacts both PAX5 and PML. A PAX5–PML fusion protein was found in the nucleus.^[50]^ This fusion inhibited PAX5 transcription in the luciferase assay and suppressed the expression of PAX5. Because PAX5 is essential for B-cell differentiation, this dominant-negative effect may block differentiation in PAX5-positive ALL. Furthermore, PAX5–PML inhibited the simulation of PML, interrupted the PML nuclear bodies, and induced apoptosis resistance in HeLa cells.^[69]^ Disruption in PML nuclear bodies, essential for lymphocyte apoptosis,^[81]^ caused acute promyelocytic leukemia cells to survive by binding to PML-RAR. So, this obstructive effect on PML induces cells of PAX5–PML-positive ALL to survive. Because of this strong negative effect on PML, PAX5-PML + ALL cells will be more likely to persist (Fig. 2).^[69]^

## 4. Conclusion

In conclusion, the discovery of PAX5 fusion in acute lymphoblastic leukemia (ALL) has shed light on the genetic basis of this deadly disease. The PAX5 gene, which is essential for B-cell development, plays a critical role in the pathogenesis of ALL when it becomes fused with other genes, resulting in the formation of abnormal proteins that disrupt normal cellular function. Identifying specific PAX5 fusion partners and their clinical implications has opened up new avenues for developing targeted therapies for ALL. The prognosis of ALL has improved over the years with advances in chemotherapy, stem cell transplantation, and supportive care. However, the prognosis remains poor for patients who relapse or are refractory to treatment, especially those with high-risk genetic features. The discovery of PAX5 fusion has provided a better understanding of the underlying molecular mechanisms of ALL and has the potential to improve clinical outcomes for patients with this disease. Several studies have shown that patients with PAX5 fusions have a distinct clinical and genetic profile compared to those without PAX5 fusions. They tend to be younger, have higher white blood cell counts, and are more likely to have additional genetic abnormalities. The identification of these features can help in risk stratification and treatment decision-making. For example, patients with PAX5 fusions have been shown to respond better to glucocorticoid therapy, which is a cornerstone of ALL treatments.

Additionally, targeted therapies such as tyrosine kinase inhibitors and immunotherapy may benefit patients with PAX5 fusions. The development of targeted therapies for PAX5 fusions in ALL is an active area of research. Several promising drugs are in preclinical and clinical development, including inhibitors of the BCR-ABL1 and JAK-STAT signaling pathways. These therapies have shown promising results in preclinical studies and early-phase clinical trials and may provide an effective and less toxic alternative to conventional chemotherapy. In conclusion, the discovery of PAX5 fusion in ALL has provided valuable insights into the genetic basis of this disease and has the potential to improve clinical outcomes for patients. Identifying specific PAX5 fusion partners and their clinical implications has opened up new avenues for targeted therapy development, which holds great promise for the future of ALL treatment. Further research is needed to fully understand the molecular mechanisms of PAX5 fusion and develop more effective and less toxic treatments for this devastating disease.

## Acknowledgments

We thank the Egypt Scholar Foundation and Prof Ahmed Abdel Aziz Bayoumi in the Faculty of Science at Cairo University for their assistance and advice.

## Author contributions

**Resources:** Fatma Mohamed Fouad.

**Supervision:** Jehane I. Eid.

**Validation:** Fatma Mohamed Fouad.

**Visualization:** Fatma Mohamed Fouad.

**Writing – original draft:** Fatma Mohamed Fouad.

**Writing – review & editing:** Jehane I. Eid.
